# Modelling of Catalytic Combustion in a Deformable Porous Burner Using a Fluid–Solid Interaction (FSI) Framework

**DOI:** 10.3390/ma16052093

**Published:** 2023-03-03

**Authors:** Tomasz Ochrymiuk, Marcin Froissart, Paweł Madejski, Janusz Badur

**Affiliations:** 1 Institute of Fluid-Flow Machinery, Polish Academy of Sciences, 14 Fiszera Street, 80-233 Gdańsk, Poland; 2 Department of Power Systems and Environmental Protection Facilities, The Faculty of Mechanical Engineering and Robotics, AGH University of Science and Technology, Mickiewicz 30 Av., 30-059 Krakow, Poland

**Keywords:** Ni_3_Al, micro combustion, catalytic combustion, surface mass, fluid–solid interaction (FSI), two-field momentum, three-fields temperature model

## Abstract

The various concepts involved in the mathematical modeling of the fluid–solid interactions (FSIs) of catalytic combustion processes occurring within a porous burner are presented and discussed in this paper. The following aspects of them are addressed: (a) the relevant physical and chemical phenomena appearing at the interface between the gas and the catalytic surface; (b) a comparison of mathematical models; (c) a proposal of a hybrid two/three-field model, (d) an estimation of the interphase transfer coefficients; (e) a discussion of the proper constitutive equations and the closure relations; and (f) a generalization of the Terzaghi concept of stresses. Selected examples of application of the models are then presented and described. Finally, a numerical verification example is presented and discussed to demonstrate the application of the proposed model.

## 1. Introduction

The science of the catalytic combustion of gases is very complex. It developed as a combination of many practical discoveries, laboratory experiments and as a result of industrial experiments. The complexity of the phenomena grew from some tremendous and intuitive prediction combinations of two components: physical responses and chemical reactions. Even fundamental characteristic parameters such as catalytic activity/inactivity, catalytic internal kinetics, the length, and the temperature of activation are variable and difficult to describe within one unified scheme.

On the other hand, modern industrial systems need compact devices for performing combustion. Therefore, in industrial practice, many different porous burners have been developed and realized. From the literature, it is well know how extremely difficult it is to describe—theoretically and numerically—the reactive flows of gases in porous media, with or without the action of catalysis. There is a lack of advanced models for describing these processes. This lack and these difficulties arise from the fact that single laboratory experiments over a solely catalytic surface cannot be directly repeated in porous media conditions. The knowledge about flows of mixtures in porous media cannot be easily and directly used in the formulation of the fundamental characteristics of combustion in porous media.

The reason for this complexity comes from the fact that the huge internal surface areas of porous media change the flow conditions of gases, both in the domain with or without chemical reactions. In particular, these difficulties come from the fact that the chemical and physical properties of the inner surfaces change during contact with different gases. Therefore, surface phenomena such as slip velocity, surface mobility, thermophoresis, and surface diffusion are different for different gases on the same surface material.

**Figure 1:**
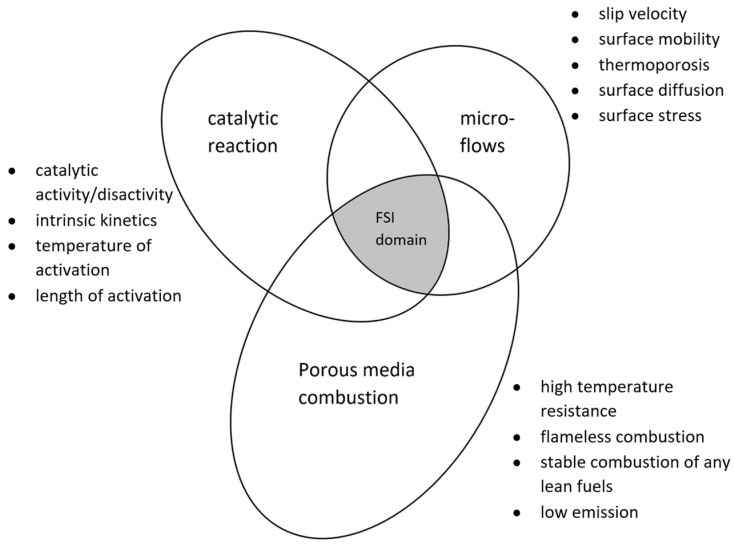
Scheme of FSI domains of interest.

Based on our experience, we recognize that three above-mentioned questions overlap within a one domain of science called fluid–solid interaction (FSI). The three main domains of scientific interest are shown schematically in Figure 1. The practical and scientific knowledge on the indicated domains has been developed very quickly, but separately, over the last two–three decades. Therefore, one can find numerous discrepancies and incoherencies when looking for more adequate mathematical models of catalytic combustion in porous burners. In particular, there is a lack of robust models when the precise mathematical modeling also needs a detailed description of the solid material, such as thermal deformation, sintering, hydration, sorption, etc. Thus, turning our attention to this problem, we supplemented the phrase “porous burner” in the title. Instead of “porous burner”, we wrote “deformable porous burner”.

Hopefully, the common framework for the integration of the three above-mentioned scientific principles into one common mathematical model is given by the concept of fluid–solid interactions (FSIs). FSIs are important in every case when the properties of thin layers of solid–fluid contacts are dominant within the whole phenomena of a model. Continua such as foams only contain a continuum of thin solid–fluid interfaces. Therefore, within the process of modeling continua with a very packed density of the interface surfaces, it is important to average the local surface properties into parameters of a bulk three-dimensional porous medium.

### 1.1. Catalytic Combustion and Experimental, Theoretical, and Numerical Research

Extensive experimental and theoretical attention has been paid to catalytic combustion over the past three decades. A lot of evidence for the significant potential of heterogeneous processes in reducing the emission pollutants, improving ignition and enhancing the stability of the generated flames has recently been recognized.

Among the many possible examples of experiments there are to take as patterns of modeling these processes, there are two baseline configurations that are often used to experimentally investigate catalytic combustion: stagnation flow fields over a catalytically active foil [1,2,3] and chemical reactors with a catalytically active wire inside them [4,5,6]. In both setups, the temperature of the catalyst is controlled by the resistive heating of the catalytic foil or wire. It is important that the mathematical modeling and simulation of such heterogeneous systems include the coupling applications between the reactive flows and the surface to capture the interactions between them at the interface. Therefore, computational tools for both systems have been recently developed, providing the ability to analyze the elementary chemical and transport processes at the gas–surface boundary and to couple them with a description of the surrounding gas phase. The aim of the undertaken efforts was to achieve a quantitative understanding of catalytic combustion (see Badur et al. [7], Badur and Ochrymiuk [8], Deutschmann [1]).

It should be noted that high embodied energy gases such as hydrogen and high-emmision gases like methane are commonly used in practice. However, recently, carbonless gases have become very important (for instance NH_3_). Therefore, from the point of view of the fundamental science, the various interactions of gases with catalysts are currently being studied at the interaction level of the gas phase molecules coming into contact with the elements of the crystal lattice of solid-phase catalysts [9].

Great progress has been achieved in this direction. In particular, phenomena such as bi-stability, surface waves, spatiotemporal chaos, patterns, chemical turbulence, and others have been studied [10].

Catalytic combustion frequently involves some reaction enhancement. An enhancement analysis was developed as an addition to the surface reaction mechanisms in order to find the reaction-controlling steps. According to practical [6,11], catalytic combustors are ceramic honeycomb-shaped devices coated with platinum or palladium. These elements are engineered to maximize the catalytic reaction—a hexagon shape ensures the maximum surface area for the minimum material usage. Strategically, catalytic combustors are designed to burn difficult or incombustible particles that are situated inside the smoke path. Catalytic combustors literally cause “difficult gases” to burn as fuel, creating more heat from the catalytically enhanced reactions. This means that dedicated fuels, such as dangerous gases, can be fully burned, transforming most of their internal energy into useful heat instead of them being released to the environment as pollution.

Yet another enhancement effect can be obtained by using perovskite catalysts with matrix-stabilized combustion in porous ceramic media [12]. If highly porous silicon carbide ceramics are used as the porous media, a catalytically enhanced super-adiabatic combustion of a lean mixture of methane and air can be easily performed. Robayo et al. [12] were also able to provide a direct regulation of the combustion flame and to obtain the effect of “perovskite catalytic enhancement of SiC” due to the use of a specially designed stainless-steel deformable chamber incorporating a quartz window.

However, from the point of view of the environmental impact of combustion, catalysis is used in the most popular catalytic converters—their application in the automotive industry has helped to reduce the emission of pollutants significantly. Special attention needs to be paid to the development of micro-catalytic combustors using high-precision ceramics. It should be noted that a catalytic burner is not a filter. Instead of trapping unburned particles, the combustor deploys chemical catalysis to break them apart. In particular, platinum and palladium atoms loaded into honeycomb-shaped cells trigger their chemical reaction at the contact surface.

A micro-scale catalytic combustor fueled by butane was investigated in the literature [13,14]. In particular, Okamasa et al. [3], developed a high-precision ceramic-tape-casting technology, which was applied in a three-dimensional combustor structure with embedded heat exchange channels. Nano-porous alumina fabricated through the anodic oxidation of aluminum layers was employed as the support for a Pd catalyst. Combustion experiments were carried out in a solder bath to keep the catalyst temperature constant. Complete fuel conversion for an n-butane flow rate of 5.0 sccm was achieved at 390 °C, corresponding to a 100 MW/m^3^ heat generation. The reaction constants for the catalytic combustion on the Pd/nano-porous alumina were determined with the aid of a 1D plug-flow model. It was also shown in a preliminary experiment in air that the reaction could be self-sustained at 425 °C with an n-butane flow rate of 15 sccm [3].

There have been a number of studies on the catalytic effects and benefits of various materials in terms of enhancing the combustion of hydrocarbon fuels. These studies start from the paper by Karim and Kibrya in 1986 who experimentally investigated the lean blowout limit of the combustion of methane and hydrogen in air [2]. The burner contained a cylindrical combustor that was 150 mm in diameter with a metallic wire mesh acting as a porous media. They coated the wire mesh with eight different materials by using an electroplating method. The materials listed in decreasing order of effectiveness are Pt, Cu, Ag, Brass, Cr, Cd, Ni, and stainless-steel 36, with the Pt coating supporting the lean combustion of methane down to 2.7% by volume, a 0.26 equivalence ratio, and the lean combustion of a 50% methane/hydrogen mixture down to a 0.15 equivalence ratio.

When comparing the obtained results against hydrogen combustion, it was found that hydrogen is more sensitive to the catalytic effects of the materials than methane, particularly at lower temperatures. In order to minimize the thermal stress at high operation temperatures, the catalyst arrangement was redesigned to the reduce temperature gradients. The surface reactions in the micro channels led to stable flames and an extremely high heat generation density of 2–3 ×10^8^ W/m^3^.

In addition, other authors (Dupont et al. [4]) investigated the stability of methane/air combustion, as well as the emission of pollutants and the radiation efficiency in a honeycomb-shaped porous media containing platinum and palladium catalysts. The honeycomb was made of cordierite and had 400 square cells per square inch. They found that the palladium catalyst supported a lower inlet concentration than the platinum and that it had a proportional effect over all the inlet flow rates tested. The results showed that the minimum stable molar concentration of CH4 was 4.4%, corresponding to 4.4 grams of Pd per piece and a 60 L/min flow rate [4].

### 1.2. Nickel Catalyst

**Figure 2:**
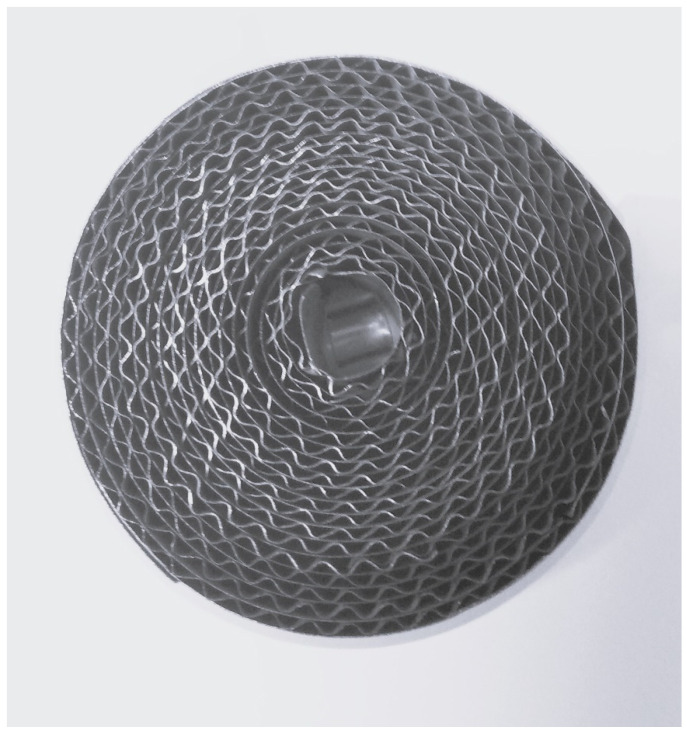
Catalytic Ni_3_Al combustor.

Palladium is not the only chemical element used as a catalyst. Many companies have based their products on nickel instead (see Figure 2). Nickel-based catalysts exhibit an extremely high catalytic activity in methanol decomposition and in the synthesis of other gasses, and they promote the production of carbon nanostructures (mainly carbon nanotubes) (Badur et al. [7], Jóźwik et al. [5]). One of the most common Ni-based solid-state catalysts is intermetallic-phase Ni_3_Al and its alloys, which belong to a family of multifunctional materials, combining the properties of both structural and functional materials. According to [15], intermetallic Ni_3_Al thin foils exhibit extremely high catalytic properties in hydrocarbon decomposition reactions.

On the other hand, the relatively high temperature required for maximal hydrocarbon conversion is the main disadvantage of this material. Nevertheless, the high temperatures of the process can be utilized by placing a regenerative heat exchanger downstream of the reactor. Jóźwik et al. [5], proposed a design for a thermo-catalytic reactor with thin strips or foils based on intermetallic-phase Ni_3_Al, which appeared as an innovative and extremely promising technology. An example of an alloy foil package based on intermetallic-phase Ni_3_Al constructed as a rolled-up sine structure is shown in Figure 2.

### 1.3. Combustion in Porous Media

It is well known that the conventional burning techniques of fuel–air mixtures have defined flammability limits, beyond which a flame cannot self-propagate due to heat losses. Porous media combustion involves two main scenarios: surface- and matrix-stabilized combustion, which are primarily defined by the flame location in each case. With surface-stabilized combustion, a flame sheet is developed on the surface of a solid porous body by many small individual laminar premixed flames. With matrix-stabilized combustion, the combustion process takes place within the solid porous media. Matrix-stabilized combustion in a porous medium is an advanced technique in which a solid porous matrix within the combustion chamber accumulates heat energy from the hot gaseous products and preheats the incoming reactants. This heat recirculation extends the standard flammability limits and allows the burning of ultra-lean fuel mixtures, conserving energy resources, or the burning of gases with a low calorific value, utilizing otherwise wasted resources. In matrix-stabilized combustion, a low-porosity inlet section is used to transfer heat from the combustion chamber to the reactants and to prevent upstream ignition. The low-porosity inlet section has a pore diameter less than the flame-quenching diameter at the operating conditions, which prevents flashback occurring, which is where the flame speed is higher than the mixture velocity and the flame propagates upstream [16].

The modelling of combustion within porous burners needs to take into account the properties of the material and to modify the momentum balance for the reacting gas mixture, and two energy balances are required for determining the two fields of temperature—the gas mixture and the solid. The optimal permeability for the preheating zone is usually identified following the so-called Carman–Cozney permeability model (Sobieski and Trykosko [17]). The modelling of pore sizes is important if the pores are smaller than the flame-quenching distance in order to prevent the flame from propagating outside of the central section (Krakowska et al. [18]).

Some studies are also available on the addition of a catalyst to a porous material for improving the properties of burners, such as studies on radiant efficiency and the emissions of existing designs. Of these, few have combined these changes with a catalyst material. With regards to materials for porous radiant combustion, both ceramic and metal compositions have been extensively explored, and a review of the most common materials is provided in the paper by Tierney and Harris [19]. For ceramics, the most common materials include cordierite, mullite, alumina, silicon carbide, zirconia or combinations of these. Ceramic materials demonstrate excellent temperature stability, making them attractive for use in porous burners [20].

### 1.4. Mathematical Modeling of Micro Flows

The main problems that are related to micro flows were described in a paper by Badur et al. [21,22,23]. The description of these phenomena includes the Stefan slip velocity, surface turbulence, surface mobility, surface jumps in temperature, and other parameters. This means that, as has been observed within experiments with a reacting gas and a catalytic surface, it is important to know the detailed relationship between the hydrodynamic and chemical processes on the catalyst surface.

Unfortunately, due to the usually complex composition of catalyst materials, it is difficult to theoretically predict the chemical reaction constants (Arrhenius and so on), and there are no publications concerning numerical research on catalysts based on, for instance, palladium-based coatings. The surface layer depends significantly on the solid temperature, on the strain rate in the near-wall boundary layer, and on the composition and temperature of the reagent flow. It is possible that at relatively low surface temperatures and low hydrogen concentrations in the external flow, heterogeneous reactions can predominate, while at higher temperatures and higher concentrations, homogeneous reactions can predominate. We refer to these phenomena as thermochemical inertia.

One example of measuring thermochemical inertia, which is governed by the time characteristic of the kinetics of surface catalysis, was performed by Kim et al. [9]. This time characteristic of catalytic kinetics on a palladium-based surface is much less than the time characteristics of convective or diffusive processes. Nevertheless, generally, the relationships between micro-flow characteristics, such as the Stefan slip velocity, and micro-catalytic reactions have not yet been determined and need further research [1].

### 1.5. FSI Framework Description

Regarding the mathematical modeling of catalytic combustion, the equations must take several phenomena into account in order to rigorously simulate a single porous channel. The key points to consider include:

(1) Heterogeneous reactions at the catalyst surface layer and homogeneous reactions in the gas phase;
(2) Mass, momentum, and thermal energy transfer by convection and diffusion in the gas phase and at the gas–solid catalytic layer;
(3) Axial heat transfer in the solid phase through the active catalyst and substrate by conduction and radiation. This assumption requires the modeling of three fields of temperature, namely the gas, solid, and catalytic layer;
(4) In addition, these phenomena are interact strongly because of the intense thermal effects associated with the heat of combustion released within the catalytic layer. These require a simultaneous simulation of porous solid thermal deformation that changes the distribution of the porosity.

The available literature has approached the phenomena of catalytic combustion from many angles (see a review by Deutschmann [1]). In the authors’ group, the so-called FSI framework has been developed for many years (Badur et al. [21,24]; Ziółkowski and Badur, [25]; Ziółkowski et al. [26]), especially for porous materials (Badur et al. [22], Ochrymiuk [27]) and thermal–FSI coupling (Froissart et al. [28,29]; Karcz and Badur [30]; Kraszewski [31], Ochrymiuk [32], Ziółkowski and Badur [33]). The great advantage of this approach is the simultaneous description of both of the unknown fields, which reduces the necessity of iteratively coupling the physical quantities between the fluid and solid.

### 1.6. Main Goals of the Article

The starting point of the presented paper is the model proposed by the authors’ group in previous articles (Badur et al. [7], Badur and Ochrymiuk [8]), which were focused on the heat transfer within a micro catalytic combustion process. This is based on a detailed chemical reaction mechanism, including a gas-phase reaction and a surface catalytic reaction. As an extension to this, a new multi-field model is proposed for adopting FSI software to simulate a real catalytic combustion process of a premixed hydrogen–oxygen flow in a sub-millimeter ceramic/alloy porous burner.

The second section describes the subject of continuum mixture formation, both in the fluid and the solid. The third section is a crucial one, because it deals with the description of the behavior of the catalytic layer in the contact zone between the fluid and solid. The equations of mass, momentum, and energy conservation within the boundary of this catalytic layer are presented. The novelty of this model is driven by the detailed modeling of the surface geometry and surface kinematics.

In Section 4, an averaged two/three-field equation for a porous catalytic combustor is presented. The concept is based on the introduction of the volumetric (not surface, so far) coefficients of mass, momentum, and energy exchange.

Section 5 contains the steps taken in developing the kinematic relationships involvedp. Section 6 describes the volumetric transport closure. Section 7 presents various new constitutive relations. Section 8, Section 9 and Section 10 are devoted to the presentation of the advanced concept of Terzaghi stresses and the concept of the material effort involved in ceramic composites.

In Section 11, the concept of taking numerical readings of disaggregated energy production is shortly presented—it has some similarity with concepts of Bejan [34], and Feidt [35,36]. Finally, the last section contains an example of an analysis based on the concept of thermal FSI.

## 2. Governing Equations

### 2.1. Evolution of Species within the Gaseous Mixture

The set of governing equations involved in this process contains the evolution of $N_{s}-1$ species, where the production rate of a species increases or decreases subject to the chemical reaction below (Badur and Ochrymiuk [8], Truesdell [37]):

(1) $$\partial_{t}\left(\varepsilon \rho Y_{i}\right)+\mathrm{div}\left(\varepsilon \rho Y_{i}v_{g}\right)=\mathrm{div}\left(J_{i}\right)+\varepsilon {\dot{\omega }}_{i}W_{i},\;i=O_{2},N_{2},CH_{4},\;H_{2},\ldots ,N_{S}-1$$

where the diffusive flux of the *i*-th species is denoted by $J_{i}$, which is defined by the Dixon–Levis law:

(2) $$J_{i}=\varepsilon \rho \;D_{mi}\;\mathrm{grad}\;\left(Y_{i}\right)$$

where $W_{i}$ is the molar density of each species. The rate of production of every species, ${\dot{\omega }}_{i}$, which is related to a single mole of the mixture, depends on the intensity of the $N_{R}$ chemical reactions, the stoichiometric coefficients $v_{ki}^{\prime }$, $v_{ki}^{\prime \prime }$, and the chemical progress coefficients $k_{k}^{f},\;k_{k}^{r}$, which depend on the Arrhenius expression (Badur and Ochrymiuk [8]):

(3) $$k_{k}^{t}=A_{k}T_{g}{}^{\beta_{k}}\exp\left(\frac{-E_{k}}{R\;T_{g}}\right),\;\;\;\;\;t=f,r$$

The activation energy, $E_{k}$, coefficients $A_{k},\;\beta_{k}$, and the stoichiometric coefficients were obtained from the chemical tables in GRI-71. The gas constant of the mixture is the sum of the species: $R=\sum_{i=1}^{N_{S}}Y_{i}R_{i}$.

### 2.2. Evolution of Species within the Composite Porous Material

Ceramic matrix composites (CMCs) are a subgroup of composite materials and a subgroup of ceramics. They consist of ceramic fibers embedded in a ceramic matrix. Both the fibers and the matrix can be manufactured from any ceramic material, including carbon and carbon fibers, which also can be regarded as a ceramic material. Recent efforts to build a better mathematical composite model have been supported by some similarities with models of mixtures of reacting gases. The first element is familiar only in terms of the physical properties of the constituents (matrix and fibers), and the sintering process is analogous to the reaction processes in gases. It is evident that a simple procedure of adding together the properties of the mixture is wrong from the very beginning, thus this analogy improves the predictions diametrically. Therefore, in the modern literature, researchers have focused on obtaining proper geometrical data before modeling the sintering process. These data can be obtained from image analysis processes obtained via scanning electron microscope (SEM) scans of the geometrical and compositional properties of the tested materials. The volume fraction of the fibers is chosen during the preparation of the material and usually makes up 1–5% of the whole volume. Regardless of the chosen experimental method, the input data for meso-mechanical analysis are not always straightforward to measure. Multi-scale strategies are helpful in generating elegant homogenized mesoscopic properties, which can be numerically identified from simulations performed at the micro-scale (Ochrymiuk [32], Orłowski et al. [38,39]).

In addition, some homogenization procedures are needed to take into account the sintering of two ceramic components. This step is much more difficult than the procedure of homogenizing heterogeneous reactions (see Equation (1)) of gases. Usually, the Mori–Tanaka model of sintering and double homogenization models for representative volumes are utilized within professional software in order to account the different physical characteristics of the composite material. Unfortunately, another factor needs to be taken into account, as compound ceramics have a distinct weak interface phase or a layered phase that is distributed throughout the bulk ceramic matrix. By varying the type and distribution of the weak interface phase or the layered phase in the composite it is possible to obtain a wide range of mechanical properties [40]. Due to limited space, this problem is not discussed in this paper; however, it must be solved within the same FSI frame.

## 3. FSI—The Governing Equations of Fluid–Solid Contact

### 3.1. The Concept of a Catalytic Surface Layer

Catalytic combustion needs the presence of a fuel–oxygen mixture contacting a the solid surface that is covered, for instance, with palladium. As experiments have shown, the combustion of hydrogen/oxygen mixtures on palladium exhibits bi-stability for lean mixtures. This happens due to the presence of a special thin gas layer that holds special physical–chemical properties, such as Stefan slip velocity [1,11,41]. Therefore, the concept of a thin catalytic interface layer formed with a special gas is correct, and so this can be used to apply the proper boundary conditions of a fluid–solid contact surface [42]. This is especially important when dealing with porous solid composite materials, where the area of the contact surface is extremely high. This paper intended to expand the range of mathematical modeling methods based on the framework of the FSI (fluid–solid interaction) approach, which describes complex continua. The basis for this further development was papers published within the Energy Conversion Department of the Institute of Fluid-Flow Machinery at the Polish Academy of Sciences (Badur et al. [23], Ziółkowski et al. [43], Ziółkowski and Badur [25], Ziółkowski et al. [26]).

The list of the main parameters characterizing catalytic surface layers includes: temperature $T_{cat}$, density $\rho_{cat}$, special surface velocity (called the Stefan slip velocity, s), and the coverage by the absorbed species $\varphi_{\left(b\right)}$. Reactive flow with catalytic properties needs to be coupled with the heterogeneous chemical reactions and the transport equations at the gas–surface interface. Therefore, masses and energy conservation equations for the species need to be established at the interface, considering a small control volume with a finite thickness of the catalytic layer.

**Figure 3:**
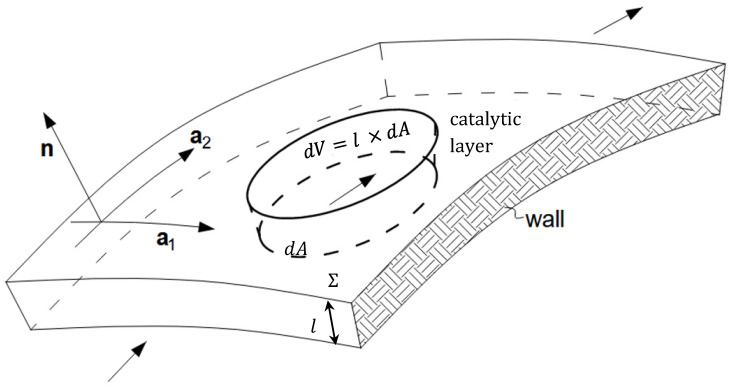
Scheme of a catalytic surface layer—the FSI framework.

The geometry of a catalytic layer is shown in Figure 3. It can be characterized by the gas layer density $\rho_{cat}$ (kg m^−2^), the particle layer velocity s (m s^−1^), and the surface momentum $\rho_{cat}s$ (kg m^−1^ s^−1^). In addition, the surface excess of the flux of momentum $t_{cat}$ can be incorporated [21].

The transport of momentum within the surface layer can be slightly different from the bulk of a typical gas mixture. For instance, it can contain recoverable (elastic) transverse components. Then, if $a_{\alpha },\;n$ (α=1,2) are the base vectors on the middle surface of the layer Σ, where n is the unit normally oriented to the surface Σ, then the surface momentum flux has complicated components:

(4) $$t_{cat}=t^{\alpha \beta }a_{\alpha }⨂a_{\beta }+t^{\alpha n}a_{\alpha }⨂n+t^{n\beta }n⨂a_{\beta }+t^{nn}n⨂n$$

This is also called the surface Cauchy stress tensor. The physical properties of the layer are unknown a priori since they depend on the resulting apparent properties of both of the contacting continua. For instance, most simple situation is the case of water contacting with air. In this case, $t_{cat}$ reduces to the surface tension, which is a two-dimensional spherical tensor tangentially oriented to the surface: $t_{cat}=\gamma I_{2}$, where γ is the water–air surface tension [kg/msm] and $I_{2}=I-n⨂n=a^{\alpha \beta }a_{\alpha }⨂a_{\beta }$ is the surface metric tensor [21,44,45]. A second metric surface tensor is usually called the curvature tensor and can be defined as the surface gradient of a normally oriented vector:

(5) $$II_{2}=-{\mathrm{grad}}_{2}\left(n\right)=-n⨂\nabla_{2}=b^{\alpha \beta }a_{\alpha }⨂a_{\beta }$$

This has the following well known variables: $I_{b}=\mathrm{tr}\left(II_{2}\right)=b_{1}^{1}+b_{2}^{2}=r_{1}^{-1}+r_{2}^{-1}$, which is the mean curvature, and $II_{b}=\det\left(II_{2}\right)=\det\left(b_{\alpha \beta }\right)=r_{1}^{-1}r_{2}^{-1}$, which is the gaussian curvature. For example, $I_{b}$ appears in the seminous Laplace formulae.

The gradient of the surface vector, for instance, the gradient of the Stefan slip velocity $s=s_{\alpha }a^{\alpha }+s_{n}n$**,** can be calculated as follows:

(6) $$\begin{array}{c} {\mathrm{grad}}_{2}\left(s\right)=\left(s_{\alpha }a^{\alpha }+s_{n}n\right)⨂\nabla_{\beta }a^{\beta }\\ =\left(s_{\alpha ;\beta }-s_{n}b_{\alpha \beta }\right)a^{\alpha }⨂a^{\beta }+\left(s^{\alpha }b_{\alpha \beta }+s_{n,\beta }\right)n⨂a^{\beta } \end{array}$$

Having obtained the surface gradient, the surface divergence can be calculated as:

(7) $${\mathrm{div}}_{2}\left(s\right)\equiv C_{1,2}\left({\mathrm{grad}}_{2}s\right)=\left(s_{\alpha ;\beta }-s_{n}b_{\alpha \beta }\right)a^{\alpha \beta }={\mathrm{div}}_{2}\left(I_{2}s\right)-I_{b}s_{n}$$

Analogically, the surface divergence [${\mathrm{div}}_{2}\;t_{cat}]$ can be calculated in the surface momentum balance. Considering the viscous flow within the surface layer, the proper rate of the surface deformation tensor must be defined as a symmetrical part of the surface gradient of the velocity, i.e., $d_{2}=0.5\left({\mathrm{grad}}_{2}\left(s\right)+{\;\mathrm{grad}}_{2}\left(s\right)\right)$. Then, the surface viscous stresses, analogously to 3D coordinates, τ=2μ d, can be written as $\tau_{2}=2\mu_{2}d_{s}$, where $\mu_{2}$ is the Navier surface viscosity coefficient [46,47]. As indicated in the literature, the Navier viscosity is a physically complex concept, since the anisotropy in the surface viscous stresses is usually experimentally observed [48,49]. This leads to the conversion of the single isotropic Navier coefficient, $\mu_{2}$, into a full constitutive expression for anisotropy. For instance, $\tau_{2\alpha \beta }=\mu_{2\alpha \beta }^{\;\gamma \delta }d_{\gamma \delta }$ (α,β,γ,δ=1,2), where $\mu_{2\alpha \beta \gamma \delta }$ is an anisotropic surface viscosity coefficient tensor [50,51,52].

### 3.2. Surface Balance of Mass

Generally, the catalytic surface density is a function of the concentration of the catalytic surface species, $Y_{\left(b\right)}$, which is governed by the developing of the species equation, i.e., Equation (18). Nevertheless, similar to the bulk stream, the total mass balance of the catalytic layer can be formulated as:

(8) $$\partial_{t}\left(\varepsilon_{s}\rho_{cat}\right)+{\mathrm{div}}_{2}\left(\varepsilon_{s}\rho_{cat}s\right)=\overset{m}{\underset{\left(b\right)}{\sum }}{\dot{m}}_{gs\left(b\right)}\left(Y_{g\left(b\right)}-Y_{s\left(b\right)}\right)$$

The sources of mass in Equation (8) cannot be taken arbitrarily; they must fulfil the mass flux fouling equation on the catalytic surface:

(9) $$\overset{m}{\underset{\left(b\right)}{\sum }}{\dot{m}}_{gs\left(b\right)}\left(Y_{g\left(b\right)}-Y_{s\left(b\right)}\right)=\overset{N_{g}}{\underset{i=0}{\sum }}{\dot{\omega }}_{i}Y_{i}$$

### 3.3. Surface Momentum Balance

The starting point for the momentum balance within the catalytic layer is quite a fundamental contact boundary condition for fluid and solid stresses:

(10) $$t_{g}n_{g}+\sigma_{s}n_{s}=0$$

where the Cauchy stress in the fluid is $t_{g}=-p_{g}I+\tau_{g}+\sigma_{g}$ and, similarly, the Cauchy stress in the solid is $\sigma_{s}=\sigma^{\prime }+\alpha p_{g}I$ (see Equations (48) and (50)). Note that $n=-n_{s}=n_{g}$.

This boundary equation can be extended by adding the surface resistivity force (Coulomb, (1801) [53]), $f_{r}=f_{r}^{Du}+f_{r}^{Na}+f_{r}^{Bu}$, which consists of three parts: the Duhem force (1898) [54], the Navier force (1822) [46], and the du Buat force (1796) [33]. These are determined via the surface viscosity coefficients. The dimensionless forms of these coefficients are called the Duhem number, the Navier number, and the du Buat number (Ziółkowski and Badur [33]). In the case of porous media, where the contact surface area is enormous, the Navier number is more important than the Reynolds number (dimensionless bulk viscosity) (Reynolds, 1901 [55]).

Furthermore, Equation (6) can be extended by incorporating the so-called surface mobility forces, $f_{m}=f_{m}^{Ga}+f_{m}^{Re}+f_{m}^{Ma}+\ldots$, that consist of several parts: the Graham surface separation force (1848) [33], the Reynolds thermal transpiration force (Reynolds, 1879 [56]), the Maxwell transpiration force (Maxwell, 1879 [57]), etc., (Badur et al. [22], Ziółkowski and Badur [25]).

Then, adding the forces within the surface control domain together, dV=l×dA (Figure 3), the following equation can be obtained:

(11) $$\left(t_{g}-\sigma_{s}\right)n+f_{r}+f_{m}=0$$

The static balance of the surface forces can be complemented with the surface divergence of the so-called Laplace tensor of momentum, $t_{cat}$ (see Equation (4)), so the static balance of the catalytic layer is governed by:

(12) $${\mathrm{div}}_{2}\left(t_{cat}\right)+\left(t_{g}-\sigma_{s}\right)n+f_{r}+f_{m}=0$$

Finally, Badur et al. [21,24], proposed a description of the whole dynamics of the surface layer with the following balance:

(13) $$\partial_{t}\left(\rho_{cat}s\right)+{\mathrm{div}}_{2}\left(\rho_{cat}s⨂s\right)+{\mathrm{div}}_{2}\left(t_{cat}\right)+\;+\left(t_{g}-\sigma_{s}\right)n+f_{r}+f_{m}+{\dot{m}}_{g}\left(v_{g}-s\right)=0$$

Using the reasoning of d’Alembert and Euler, the definition of a surface acceleration vector is as follows (D’Alembert [58]):

(14) $$a_{2}=\frac{d_{2}}{dt}s=\partial_{t}s+\left({\mathrm{grad}}_{2}s\right)(I_{2}s)$$

The momentum balance of the layer (Equation (10)) becomes a nonlinear differential equation for two additional unknown fields: the surface mass density $\rho_{cat}$ and the layer slip velocity. If $t_{cat}=\gamma I_{2}$, $t_{g}=p_{g}I$, $\sigma_{s}=p_{s}I$, then the layer momentum (Equation (9)) leads to the generalized Young–Laplace equation [47,59,60]:

(15) $${\mathrm{div}}_{2}\left(\gamma I_{2}\right)+\left(p_{g}-p_{s}\right)n=0$$

In general, the flux of the layer momentum, $t_{cat}$ (see Equation (13)), is responsible for the recoverable (elastic) and viscous transport of the surface momentum, i.e., $t_{cat}=t_{cat}^{\left(e\right)}+t_{cat}^{\left(v\right)}$. The first and most important part of elastic flux is known as the capillarity diade, $\gamma I_{2}$. However, Stokes, in 1845, introduced an additional “normal” part, ϖ, and in 1876, Gibbs added the curvature tension part, C, such that [23,44]:

(16) $$t_{cat}^{\left(e\right)}=\gamma I_{2}+CII_{2}+\varpi n⨂n$$

The viscous properties of the catalytic layer depend on the so-called “apparent viscosity”, which, in general, possesses a transversal anisotropy. Using the diade of the surface deformation rate, $d_{2}=\frac{1}{2}\left({\mathrm{grad}}_{2}\left(s\right)+{\;\mathrm{grad}}_{2}\left(s\right)\right)$, the following viscous stress tensor can be obtained [25]:

(17) $$t_{cat}^{\left(v\right)}=\lambda^{\prime }\left(tr\;d_{2}\right)I_{2}+\lambda^{\prime \prime }\left(s_{n,n}\right)n⨂n+2\mu^{\prime }I_{2}d_{2}I_{2}+2\mu^{\prime \prime }\left(d_{2}-I_{2}d_{2}I_{2}\right)$$

where the formulas for the four apparent surface viscosities, $\lambda^{\prime },\;\lambda^{\prime \prime },\;\mu^{\prime },\;and\;\mu \prime \prime$, need further investigation. Nevertheless, these coefficients should be distinguished from the surface viscosity such as the Navier surface viscosity. See the papers by Arkilic et al. [42], Kowalewski et al. [61], Lockerby et al. [52] and Morini et al. [59], where fundamental experiments were developed.

### 3.4. Catalytic Evolution of Species

The conditions of the catalytic surface are described by the temperature and the coverage with the adsorbed species. The reactive flow has to be coupled with the heterogeneous chemical reactions and the transport at the gas–surface interface. Therefore, conservation equations for species masses and energy can be established at the interface, considering a small control volume of the catalytic layer, dV=l×dA (Figure 3, adjacent to the surface. Then, the mass fraction of a gas-phase species $\left(b\right)$ at the surface is determined by the diffusive and convective processes and the production or depletion rate $\left(b\right)$ of a given species by surface reactions:

(18) $$\partial_{t}\left(\varepsilon_{s}\rho_{cat}Y_{\left(b\right)}\right)+{\mathrm{div}}_{2}\left(\varepsilon_{s}\rho_{cat}Y_{\left(b\right)}s\right)=\;={\mathrm{div}}_{2}\left(j_{\left(b\right)}\right)+\left[J_{\left(b\right)}+\rho_{cat}sY_{\left(b\right)}\right]∙n+{\dot{m}}_{gs\left(b\right)}\left(Y_{g\left(b\right)}-Y_{s\left(b\right)}\right)+\varepsilon_{s}{\dot{\omega }}_{\left(b\right)}W_{\left(b\right)}\;\mathrm{for}\;\left(b\right)=1,2,3,\ldots$$

where $\rho_{cat}$ is the surface density [kg m^-2^] of the catalytic layer, $Y_{\left(b\right)}$ is the mass fraction of a species in the control layer, $j_{\left(b\right)}$ is the surface diffusive flux (including thermal diffusion), $J_{\left(b\right)}=J_{i}$ for $\left(b\right)\equiv i$ species is the mass flux diffusion of species i, as discussed in Equation (1). The surface porosity $\varepsilon_{s}$ is defined as the ratio of the heterogeneous surface reaction area $A_{cat}$ (desorption minus adsorption) to the unit surface area $A_{geo}$:

(19) $$\varepsilon_{s}=A_{cat}/A_{geo}$$

The area, $A_{cat}$, refers to the actual catalytically active surface area and can be determined experimentally, e.g., by chemisorption measurements [14]. The ratio $\varepsilon_{s}$ is also used to describe the dependence of the overall reaction rate on catalyst loading and the effects of hydro-thermal aging for structure-insensitive catalysts [62]. It was recently applied to model the performance of on-road-aged three-way catalysts [63].

The surface porosity is very similar to the so-called surface coverage factor $\varphi_{\left(b\right)}$ i.e., the fraction of the surface sites covered by species $Y_{\left(b\right)}$. The rate of change $\varphi_{\left(b\right)}$ depends on ${\dot{\omega }}_{\left(b\right)}$ in a simple manner, as described in the paper by Deutschmann [1]:

(20) $$\partial_{t}\varphi_{\left(b\right)}=\beta {\dot{\omega }}_{\left(b\right)}\;\mathrm{for}\;\left(b\right)=1,\;2,\;3,\ldots m$$

where β is the surface site density of the catalyst. The numerical solution of the catalytic evolution of a species (Section 3.4) must be performed. As the surface momentum balance (Section 3.3) and the catalytic evolution of species (Section 3.4) are fully time-dependent, transient phenomena such as ignition, oscillation, extinction, or catalyst poisoning can be described in detail. The surface coverage also fulfills the site fraction conservation law, $\overset{m}{\underset{\left(b\right)=1}{\sum }}\varphi_{\left(b\right)}=1$.

Returning to Equation (18), the term ${\dot{\omega }}_{\left(b\right)}W_{\left(b\right)}$ indicates the source of the heterogeneous surface reaction rate (desorption minus adsorption), which is given in kg m^−2^ s^−1^. The molar net production rate of gas phase species b given as ${\dot{\omega }}_{\left(b\right)}$ (mol m^−2^ s^−1^) now refers to $W_{\left(b\right)}$, which is the actual molecular mass of species b; m is the number of gas-phase species on the surface, and dA is the surface layer area. Additionally, in Section 3.4, the outward-pointing unit vector n is normal to the surface, so if chemical surface reactions occur, the slip velocity vector s can be non-zero at the catalytic surface.

This so-called Stefan slip velocity, s, which is given by the temperature of the catalyst, is derived from various contributors to the energy balance at the catalyst layer. A non-zero Stefan velocity, s**,** occurs for the net mass flux between the surface and the gas phase [1,64]:

(21) $$s\cdot n=\sum_{\left(b\right)=1}^{m}\varepsilon_{s}{\dot{\omega }}_{\left(b\right)}W_{\left(b\right)}\;$$

At steady-state conditions, this mass slip disappears unless mass is either deposited on the surface (e.g., chemical vapor deposition) or ablated (e.g., material etching). Equation (6) basically means that for s=0, the amount of gas-phase molecules of species b, which are consumed/produced at the catalyst by adsorption/desorption, have to diffuse to/from the catalytic wall (Equation (7)). Only for fast transient adsorption/desorption processes, e.g., during the ignition of catalytic oxidation, does the steady-state Equation (6) break down and special treatment of the coupling is needed. Furthermore, these fast transient processes may lead to heat accumulation terms as well as to additional convective transport and the associated pressure gradients in the fluid phase above the catalyst [62].

Concerning ${\dot{\omega }}_{\left(b\right)}$, it is known that the rate of a catalytic reaction is very specific to the catalyst formulation; therefore, only global rate expressions have been used for many years [1,3]. These reaction rates are based on the catalyst mass, catalyst volume, reactor volume, and the catalyst external surface area. The implementation of this surface-kinetics-based approach into mathematical simulations is straightforward; the reaction rate can, in general, be expressed by any arbitrary function of gas-phase concentrations, and the temperature at the catalyst surface, $T_{cat}$, is calculated at every computational cell containing either catalytically active particles or walls.

It is evident that this catalytic-layer-like approach cannot account for the complex variety of phenomena of catalysis and that the rate parameters must be evaluated. The surface chemistry is modeled by elementary reactions on a molecular level in the gas phase and in the solid molecules at the surface. As usual, the temperature dependence of the reaction rate can be described by a modified Arrhenius equation. Special care must be taken for the additional coverage dependence of the rate of some surface reactions (Badur et al. [7]). For reversible reactions, the rate coefficients are related to the forward rate coefficients through the equilibrium constant. For instance, the reactions of hydrogen, oxygen, and methane on polycrystalline platinum along with their rate expressions are given explicitly in the paper by Kang et al. [63]. All the pre-exponential factors, A, were chosen to be independent of the temperature. Details on the reaction steps and the rate data are discussed elsewhere (Badur and Ochrymiuk [8], Weisz [14]). The thermochemical data needed to calculate the equilibrium constants for the reversible reactions were taken from [65].

It should be highlighted that the correct and direct mathematical modeling of surface catalytic reactions has up to now been too difficult to be conducted. It is obvious that the direct computation of the chemistry of surface reaction rates at the molecular level leads to a closer comprehensive description, at least for idealized systems. However, powerful methods of mathematical modeling such as direct numerical simulations (DNS), large eddy simulations (LES), lattice Boltzmann models (LBM), density functional theory (DFT), molecular dynamics (MD), and the Monte Carlo (MC) method are currently only in the initial and development stages. Therefore, these approaches cannot be implemented in a complex simulation of flow/deformation fields undergoing a catalytic reaction of technically relevant systems. This is due to a lack of efficient algorithms that are able to reduce the immense amount of computational time currently needed (Deutschmann [1]).

In the presented FSI framework approach, the catalytic system is treated as a black box. There is no alternative; the knowledge gained from experimental and theoretical surface science studies can be implemented in the chemical models used in reaction engineering simulations. A tractable approach involves the treatment of surface chemistry by rate equations that are strongly governed by the surface catalytic temperature $T_{cat}$ and a set of surface coverage factors, $\varphi_{\left(b\right)}$ (see Equation (6)). They depend on the time and the meso/macroscopic reaction position, but they are averaged over microscopic local fluctuations. The surface structure of the catalyst is associated with a surface site density, β, that describes the maximum number of species adsorbing on a unit surface area, given in mol m^-2^. Each surface species, $Y_{\left(b\right)}$, is associated with a coverage, $\varphi_{\left(b\right)}$. Under the assumptions made, a multi-step (quasi-elementary) reaction mechanism can be set up. The local chemical molar source term is then defined by the following equation (Badur and Ochrymiuk [8]):

(22) $${\dot{\omega }}_{\left(b\right)}=\sum_{p=1}^{N_{s}}\nu_{\left(b\right)p}k_{p}\overset{N_{s}+N_{r}}{\underset{\left(j\right)=1}{\prod }}{\left(X_{\left(j\right)}\right)}^{\nu_{\left(j\right)p}^{\prime }}$$

where $N_{s}$ is the number of surface reactions, $\left(X_{\left(j\right)}\right)$ is the molar surface concentration (mol m^−2^), and $\nu_{\left(b\right)p}$ and $\nu_{\left(j\right)p}^{\prime }$ are the stoichiometric coefficients. The expression for the rate coefficient, $k_{p}$, is still in the research stage, although in the literature there are numerous chemical hypotheses concerning it. The most popular is given by Equation (13) (Badur and Ochrymiuk [8]):

(23) $$k_{p}=A_{p}{\left(T_{cat}\right)}^{\beta_{p}}\;\exp\left[\frac{E_{p}}{RT_{cat}}\right]\;\overset{m}{\underset{\left(b\right)=1}{\prod }}{\left(\varphi_{\left(b\right)}\right)}^{\xi_{\left(b\right)}}\exp\left[\frac{e_{\left(b\right)}\varphi_{\left(b\right)}}{RT_{cat}}\right]$$

where two additional coverage dependence factors, $\xi_{\left(b\right)}$ and $e_{\left(b\right)}$, appear (Prasad et al. [13]). Here, $A_{p}$ is the pre-exponential factor, $\beta_{p}$ is the temperature exponent, and $E_{p}$ is the activation energy. It should be remembered that thermodynamic data for adsorbed species are difficult to measure, so the thermodynamic consistency of surface kinetics for reaction networks is a crucial issue. Two alternative methods have been proposed to enforce thermodynamic consistency without the necessity of explicitly knowing all the thermodynamic adsorbents [13,41].

### 3.5. Catalyst Temperature—Surface Energy Balance

The temperature of a catalytic reaction, $T_{cat}$, can be determined from a surface energy balance. The key assumption taken forward is that the catalytic layer is able to perform conductive, convective, and diffusive energy transport from the gas phase adjacent to the surface. This includes the chemical heat released at the surface, the thermal radiation, and the resistive heating at the catalyst surface. This assumptions results in the following surface energy equation [33]:

(24) $$\partial_{t}\left(\rho_{cat}c_{cat}T_{cat}\right)+{\mathrm{div}}_{2}\left(\rho_{cat}c_{cat}s\right)-T_{cat}I_{2}sn+{\mathrm{div}}_{2}\left(j_{cat}\right)+h\left(T_{g}-T_{s}\right)+\;+\left(q_{g}-q_{s}\right)\cdot n+\sum_{i=1}^{N_{s}}h_{i}\left(J_{i}+\rho_{g}Y_{i}s\right)\cdot n+\sigma_{em}\varepsilon_{em}\left(T_{cat}^{4}-T_{ref,c}^{4}\right)+\;+\overset{m}{\underset{\left(b\right)=1}{\sum }}{\dot{\omega }}_{\left(b\right)}W_{\left(b\right)}h_{\left(b\right)}+f\cdot s+I^{2}R=0$$

where s is the Stefan catalytic slip velocity, $f=f_{r}+f_{m}\;c_{cat}$ denotes the specific heat capacity of the catalyst, and $\rho_{cat}$ is the density of the catalyst layer. The surface heat flux, $j_{cat}$, is given by an analogy to the Fourier conductivity law, $j_{cat}=\lambda_{cat}{\mathrm{grad}}_{2}(T_{cat})$, and $h_{\left(b\right)}$ is the specific enthalpy of the catalytic species b, either in the gas phase or at the surface. In the radiation term, $\sigma_{em}$ is the Stefan–Boltzmann constant, $\varepsilon_{em}$ the temperature-dependent surface emissivity, and $T_{ref,c}$ is the reference temperature at which the surface radiates. The term f·s represents the contribution of specific surface work [66].

Additionally, the term $I^{2}R$ represents an energy source corresponding to the resistive heating of the catalyst, where I is the current and R is the electrical resistance, depending on the temperature. In summary, m is the number of surface species, $c_{p}$ is the specific heat capacity of the gas at the wall, and h denotes a heat transfer coefficient that includes the catalyst volume and a small control volume in the gas phase adjacent to the surface.

## 4. A Set of the Two-Component Continuum Governing Equations

Once the independent balance equations for gases and solids within the bulk and on the moving boundary (so-called FSI boundary conditions) have been obtained, an efficient model of a porous, two-component continuum where the gas and solid surface take an equivalent role can be constructed. It is obvious that the surface boundary conditions, where divergence [${\mathrm{div}}_{2}\left(\cdot \right)]$ and surface gradients [${\mathrm{grad}}_{2}\left(\cdot \right)$] (see Equations (5) and (7)) appear, cannot be further useable in a three-dimensional “equivalent” continuum. Therefore, the dense contributions of those ${\mathrm{div}}_{2}\left(\cdot \right)$ and ${\mathrm{grad}}_{2}\left(\cdot \right)$ terms multiplied by the surface density a should be replaced by some “internal closures” that can be interpreted as some internal sources that come from the internal substructure of the continuum mixture.

In order to consider the catalytic combustion process within a porous burner made of modern composites (assuming that the catalytic effect of the porous material at high temperatures is not negligible), any simplification within the porosity effect is a rather wrong assumption, since the “heart” of porous burners are catalytic reactions. The geometry of porous composites can be determined by the following parameters [18]:

- ε—porosity (m^3^/m^3^), typical values between 0.7 and 0.9;
- a− pore surface density (m^2^/m^3^), typical values between 1 and 200;
- τ—tortuosity (m/m), typical values between 1.1 and 2.0.

Please note that in the literature on porous materials [67,68], two-field models dominate.

In the calculation domain, two coordinates dominate, namely x and r, or axial and radial, as the circumferential direction in the first approach can be omitted. The porosity of a burner, ε, typically has values of ε=0.7−0.9. $T_{g}$ denotes the temperature field of the gases and $T_{s}$ denotes the temperature of the solid material. In this paper, the well-established European denotations “grad” and “div” are used instead of “nabla”.

**Figure 4:**
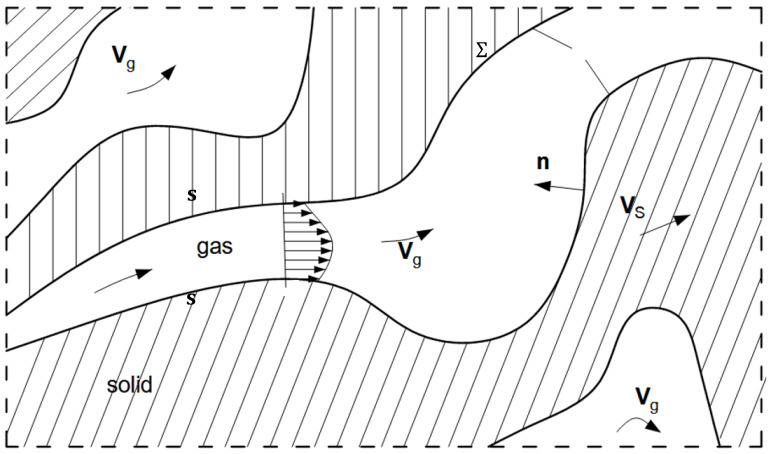
Scheme of porous continua filled with gas catalytic combustion.

The baseline assumption is that the porous mixture possesses a two-component (see Figure 4) type of modelling, namely gas $\left(g\right)$ and solid $\left(s\right)$. As a result, a set of mass, momentum, and energy balances is dedicated to each component.

### 4.1. Mass Balance

Within the framework of two-field density, two-field momentum, and three-field temperature, there is no more room in the catalytic layer Equation (8). Therefore, in this approach, the transport of mass is governed by a volumetric (bulk) coefficient, ${\dot{m}}_{gs\left(b\right)}$.

#### 4.1.1. Total Mass Balance

The mass balance of the gaseous mixture takes a similar form to the continuity equation of the reacting mixture:

(25) $$\partial_{t}\left(\varepsilon \varrho_{g}\right)+\mathrm{div}\left(\varepsilon \rho_{g}v_{g}\right)=\sum_{\left(b\right)=1}^{M}{\dot{m}}_{gs\left(b\right)}\left(Y_{g\left(b\right)}-Y_{s\left(b\right)}\right)$$

the porous material under deformation is given as:

(26) $$\partial_{t}\left(\left(1-\varepsilon \right)\varrho_{s}\right)+\mathrm{div}\left(\left(1-\varepsilon \right)\rho_{s}v_{s}\right)=-\sum_{\left(b\right)=1}^{M}{\dot{m}}_{gs\left(b\right)}\left(Y_{g\left(b\right)}-Y_{s\left(b\right)}\right)$$

where the velocity of the gas mixture is $v_{g}$, and the velocity of the solid is $v_{g}=\partial_{t}u=\dot{u}$, where u denotes the displacement field of the solid composite. The explicit formula for the density of the gas mixture, $\rho_{g}$, uses the mass, $Y_{i}$, or volume fraction of species $X_{i}$ for expressing $\rho_{g}=\sum_{i=1}^{N_{S}}Y_{i}\rho_{i}$. The index $i=O_{2},N_{2},CH_{4},\;H_{2},\ldots ,N_{S}$ deals with the mixture of the species, and $N_{S}$ indicates how many species there are.

The total transport of mass between the fluid and solid is the sum of $\left(b\right)=1,\;2,\;3,\;\ldots ,\;m$ surface components and the volumetric transport coefficients, ${\dot{m}}_{gs\left(b\right)}$.

Concerning the change in the porosity with time, $\partial_{t}\varepsilon =?$, there are a few strategies to solve this problem [69]. Some researchers use quite an independent evolution equation for the porosity. In the present approach, since composite materials can be strongly deformed, the change in the porosity can be described by an additional “closure” equation, i.e., Equation (44). Additionally, it was assumed that the pore surface density, a, and the tortuosity, τ, are constant.

#### 4.1.2. Evolution of Species in Reacting Gases

The composition of the homogeneous and heterogeneous evolutions of $N_{R}+m$ species within the reacting mixture is given in Equations (1) and (18). However, these equations cannot be simply added, since Equation (18) must be multiplied by the surface density, a. Therefore, the set of governing equations must also contain the homogeneous and heterogeneous (catalytic) evolution for $N_{s}-1$ species, where the rate of the increase/decrease in the species content mainly depends on chemical reactions:

(27) $$\partial_{t}\left(\varepsilon \rho Y_{i}+a\varepsilon_{s}\rho_{cat}Y_{\left(i-m\right)}\right)+\mathrm{div}\left(\varepsilon \rho Y_{i}v_{g}+a\varepsilon_{s}\rho_{cat}Y_{\left(i-m\right)}s\right)=\;=\mathrm{div}\left(J_{i}+aj_{\left(i-m\right)}\right)+\varepsilon {\dot{\omega }}_{i}W_{i}+\varepsilon_{a}a{\dot{\omega }}_{\left(i-m\right)}W_{\left(i-m\right)}+{\dot{m}}_{gs\;\left(i\right)\;}\left(Y_{i}-Y_{\left(i-m\right)}\right)\;\mathrm{for}\;i=1,2,3,\ldots ,N_{s}+m-1$$

where $Y_{i}$ (kg/kg) is the mass fraction of species $i=O_{2},N_{2},CH_{4},\;H_{2},\ldots ,N_{S}$, which takes into account not only the bulk reaction but also the surface catalytic reaction. $J_{i}$ and $J_{\left(i-m\right)}$ are the Dixon–Levis diffusive flux bulk and surface transport, and $W_{i}$ is the molar density of each species. In addition, in Equation (27), the rate of the production of every species, ${\dot{\omega }}_{i}$, is related to the number of moles of the mixture.

### 4.2. Momentum Balance

A two-component continuum particle possesses two momentum balance equations, one to determine the gas velocity $v_{g}$ and the second to determine the solid velocity $v_{s}$:

(28) $$\partial_{t}\left(\varepsilon \rho_{g}v_{g}\right)+\mathrm{div}\left(\varepsilon \rho_{g}v_{g}⨂v_{g}\right)=\mathrm{div}\left(\tau_{g}-p_{g}I+\sigma_{g}\right)+b_{gs}\left(v_{g}-v_{s}\right)$$

(29) $$\partial_{t}\left(\left(1-\varepsilon \right)\rho_{s}v_{s}\right)+\mathrm{div}\left(\left(1-\varepsilon \right)\rho_{s}v_{s}⨂v_{s}\right)=-\mathrm{div}\left(\sigma_{s}-p_{g}I\right)-b_{gs}\left(v_{g}-v_{s}\right)$$

In the above (six-scalar) Equations (28) and (29), ε is the porosity, $\rho_{g}\;and\;\rho_{s}$ are the densities of the mixture of gases and solid composites, $v_{g}\;and\;v_{s}$ are the velocity of the gas mixture and the solid, $\tau_{g}$ is the total viscous stress tensor in the gas mixture, $p_{g}$ is the pressure of the gas mixture calculated based on the Dalton assumption about the summation of partial pressures of gas components, $b_{gs}\left(v_{g}-v_{s}\right)$ is the fluid–solid momentum interaction determined by the local coefficient of interaction $b_{gs}$, $\sigma_{s}$ is the partial stress tensor at the solid composite, and, finally, $\sigma_{terz,s}=\sigma_{s}-p_{g}I$ is the so-called effective Terzaghi stress tensor. The direct presence of the fluid pressure, $p_{g}$, in the solid momentum balance is due to some freedom of fluid penetration inside the solid; however. such a directly symmetrical influence of the solid on the momentum flux tensor does not yet exist. Therefore, the introduction of the term $\sigma_{g}$**,** which is a recoverable, elastic-like stress tensor in the gas component, comes from the fluid–solid interaction. By analogy, the term $t_{terz,g}=\tau_{g}-p_{g}I+\sigma_{g}$ is called the effective Terzaghi stress tensor in the bulk gas. Note that in Equations (28) and (29), classical body forces were omitted.

There is a special momentum balances case when a solid is deformable but stationary ($v_{s}=0$). leading to a special form of Equations (28) and (29). The momentum balance in the gas mixture of the continuum is given as follows:

(30) $$\partial_{t}\left(\varepsilon \rho_{g}v_{g}\right)+\mathrm{div}\left(\varepsilon \rho_{g}v_{g}⨂v_{g}\right)=\mathrm{div}\left(\tau_{g}-p_{g}I\right)+f_{D-F}$$

where the resultant force $f_{D-F}$ is called the Darcy–Forchheimer slip resistance force (sometimes called “the pressure drop”) (Moghaddam and Jamiolahmady [70]). It is given as:

(31) $$f_{D-F}=\frac{\mu }{K_{1}}\;\varepsilon v_{g}+\frac{\mu }{K_{2}}\;\varepsilon^{2}\left|v_{g}\right|v_{g}$$

where μ is the bulk shear viscosity of the mixture, and $K_{1}\;and\;K_{2}$ are the Darcy–Forchheimer slip resistance coefficients (Moghaddam and Jamiolahmady [70], Sobieski and Trykozko [17]).

In the literature, together with fluid momentum balance, there is also a fluid porosity balance, which is written as an evolution equation for porosity ε:

(32) $$\partial_{t}\left(\rho_{g}\varepsilon \right)+\mathrm{div}\left(\rho_{g}j_{D}\right)=J$$

where $\rho_{g}$ is the fluid density, ε is the porosity, $j_{D}$ (a vector quantity) is the Darcy flux, or more specifically, the flux of the pore fluid. The first term in Equation (31) describes the changes in the fluid stored in the control volume, and the second term describes the net fluid flux across the control volume faces. In the above, J is a fluid source or sink, which may be either at discrete locations or distributed in an arbitrary fashion throughout the domain. It is an open question how to derive the Darcy flux, $j_{D}$, because some force equilibrium conditions apply to the fluid as well as the porous medium. Constitutive closure is manifested through a relationship between the fluid flux and the forces driving the flux. For instance, for nearly all geological applications, the appropriate constitutive relationship is Darcy law, which can be expressed as (Krakowska et al. [18]):

(33) $$j_{D}=-k\left(\frac{1}{\mu_{g}}\right)\left[\mathrm{grad}\;p_{g}+\varrho_{g}g\;\mathrm{grad}\;\psi +p_{g}\;\mathrm{grad}\left(I_{\varepsilon }\right)\;\right]$$

where $k=k_{ml}e_{m}⨂e_{l}$ is a second-order permeability tensor, $\mu_{g}$ is the fluid dynamic viscosity, g is the gravitational acceleration, ψ is the gravitational potential (elevation above an arbitrary datum), and $I_{\varepsilon }=\mathrm{tr}\;\left(\varepsilon \right)$ is the trace of the solid deformation tensor. Darcy’s law shows that the porosity change is a function of the flow fluxes driven by gradients in terms of pressure energy, deformation energy, and elevation energy. In the undertaken approach, it was assumed that the porosity only changes due to deformation.

The static momentum balance in a porous solid (Equation (29)) reduces to the form:

(34) $$\mathrm{div}\left(\sigma_{s}-p_{g}I\right)-f_{D-F}=0$$

### 4.3. Three-Field Energy Balance

According to the concept of three temperature fields, every particle control volume needs three independent energy balance equations for all the unknowns:

#### 4.3.1. Energy Balance in the Gas Constituent

The energy balance in the gas constituent is related to the internal energy balance of the gas mixture, $\epsilon =c_{p}T_{g}$, in the following form:

(35) $$\partial_{t}\left(\varepsilon \rho_{g}\epsilon \right)+\mathrm{div}\left(\varepsilon \rho \epsilon v_{g}\right)=\mathrm{div}\;\left(\varepsilon q_{g}\right)+h_{cat}\left(T_{cat}-T_{g}\right)-\varepsilon \;\left(\sum_{k=1}^{N_{R}}{\dot{\omega }}_{k}h_{k}\right)$$

In the three-temperature model, $T_{cat}$, $T_{s}$, and $T_{g}$, there are three interpenetrating temperatures in the continuum volume that cannot be simply mixed and equilibrated. Therefore, a “volumetric” heat exchange coefficient, $h_{cat}$, needs to be introduced. Note that in the first step, the contribution due to the surface catalytic temperature can be represented as a surface source: $h_{cat}\left(T_{cat}-T_{s}\right)≅\overset{m}{\underset{\left(b\right)=1}{\sum }}{\dot{\omega }}_{\left(b\right)}W_{\left(b\right)}h_{\left(b\right)}$.

Equation (35) also contains classical parts that are additionally equilibrated with an internal energy exchange between the fluid and solid. There are two types of sources, one due to chemical reactions in the bulk gas, $N_{R}$, and a second due to the catalytic reactions on the surface, $N_{c}$. In this equation, the mixture density and heat capacity can be defined as:

(36) $$\rho_{g}=\sum_{i=1}^{N_{S}}Y_{i}\rho_{i},\;\;\;\;c_{p}=\sum_{i=1}^{N_{S}}Y_{i}c_{p,i}$$

The rate of the bulk gas reaction is denoted as ${\dot{\omega }}_{k}$, and the enthalpy of the reaction is given by $h_{k}$. Similarly, the rate of the catalytic reaction at the surface is denoted as ${\dot{\omega }}_{\left(b\right)}$, and the enthalpy of the catalytic reaction is given by $h_{\left(b\right)}$.

#### 4.3.2. Energy Balance in the Solid Constituent

The energy balance in the solid constituent takes into account heat diffusion flow, volumetric heat exchange, and energy radiation to the environment. It is given as:

(37) $$\partial_{t}\left(\left(1-\varepsilon \right)\rho_{s}\epsilon_{s}\right)=\mathrm{div}\;\left(\left(1-\varepsilon \right)q_{s}\right)-h_{cat}\left(T_{cat}-T_{s}\right)-{\dot{Q}}_{rad}$$

where the solid energy density is $\epsilon_{s}=c_{p}T_{s}$, $q_{s}$ is the heat flux in the solid, and the main source of energy is emitted through radiation, ${\dot{Q}}_{rad}$ [J s^−1^ m^−2^].

#### 4.3.3. Energy Balance in the Catalytic Layer in Terms of Temperature

In the multi-field concept of a continuum, the distribution of the catalytic temperature, $T_{cat}$, cannot be established directly from Equation (24) due to the too many contact faces between the gas and the surface of the porous media. As a result, some averaging technique needs to be applied. Assuming that the surface density is constant, a=const, Equation (24) can be multiplied by the surface density to level-off the dimensions and spear the field of the catalytic temperature. Under these assumptions, it transforms into the following surface-like energy equation:

(38) $$\partial_{t}\left(a\rho_{cat}c_{cat}T_{cat}\right)+\mathrm{div}\left(a\rho_{cat}c_{cat}s\right)+\mathrm{div}\left(aj_{cat}\right)+h_{cat}\left(T_{g}-T_{s}\right)+\;+\sigma_{em}\varepsilon_{em}\left(T_{cat}^{4}-T_{ref,c}^{4}\right)+\overset{m}{\underset{\left(b\right)=1}{\sum }}{\dot{\omega }}_{\left(b\right)}W_{\left(b\right)}h_{\left(b\right)}=0$$

where s is the Stefan catalytic slip velocity, $c_{cat}$ denotes the specific heat capacity of the catalyst, and $\rho_{cat}$ is the density of the catalyst layer. The surface-like heat flux, $j_{cat}$, is given by an analogy to the Fourier conductivity law, i.e., $j_{cat}=\lambda_{cat}\mathrm{grad}(T_{cat})$, and $h_{\left(b\right)}$ is the specific enthalpy of the catalytic species b, either in the gas phase or at the surface. In the radiation term, $\sigma_{em}$ is the Stefan–Boltzmann constant, $\varepsilon_{em}$ is the temperature-dependent surface emissivity, and $T_{ref,c}$ is the reference temperature at which the surface radiates.

The volumetric heat exchange coefficient, $h_{cat}$, is the same as in Equations (35) and (37), allowing a direct connection of the three temperature fields.

## 5. Kinematic Relationships

Kinematic relationships are based on a continuum divided into particles containing the following basic unknowns: $N_{s}$ fields of the mass fraction of species $Y_{i}$, $N_{s}$ fields of the mass fraction of the surface species $Y_{\left(b\right)}$, the actual solid density, two fields of velocity $v_{g}$ and $v_{s}$, and three fields of temperature, $T_{cat}$, $T_{s}$, and $T_{g}$. Therefore, the kinematic relationships are as simple as possible. The most important parameter is the tensor of the rate of fluid deformation, which is defined as a symmetrical part of the velocity gradient:

(39) $$d=\frac{1}{2}\;\left(\mathrm{grad}\;v_{g}+{\mathrm{grad}}^{T}\;v_{g}\right)=d_{jk}e_{j}⨂e_{k}$$

where the first invariant is equal to $I_{d}=\mathrm{tr}\left(d\right)=\mathrm{div}\;v_{g}$. For solids, this kinematical relationship looks similar. The rate of solid deformation is given as:

(40) $$\dot{\varepsilon }=\frac{1}{2}\;\left(\mathrm{grad}\;v_{s}+{\mathrm{grad}}^{T}\;v_{s}\right)={\dot{\varepsilon }}_{jk}e_{j}⨂e_{k}$$

where the small deformation tensor, ε**,** is based on solid displacement. The gradient of temperature fields is used in the fluid and solid sub-domains, for instance:

(41) $$g_{g}=\mathrm{grad}\;T_{g}\;\mathrm{and}\;\;g_{s}=\mathrm{grad}\;T_{s}$$

where $g_{g}$ and $\;g_{s}$ are the temperature gradients in the fluid and solid, respectively.

## 6. Constitutive Relationship for the Interaction Coefficients

The presented model is based on three main coefficients, namely ${\dot{m}}_{gs\left(i\right)}$, $b_{gs}$, and $h_{cat}$ (appearing in Section 4.3), which are the volumetric mass transport, the volumetric momentum transport, and the volumetric heat exchange coefficient, respectively. All of them have not yet been determined in this paper.

### 6.1. Volumetric Mass Transport

The volumetric mass transport coefficient is responsible for the mass jump related with the Lewis jump of the concentration. It is correlated with the surface jump coefficient $L_{\left(i\right)}$ (Groppi et al. [11]), which is individually measured for every species’ mass concentration (fraction):

(42) $${\dot{m}}_{gs\left(i\right)}=a\varphi_{\left(i\right)}L_{\left(i\right)}\;\mathrm{for}\;i=\left(b\right)\;$$

where a is the surface volumetric density, and $\varphi_{\left(i\right)}$ is the site fraction (Equation (20)).

### 6.2. Volumetric Momentum Transport

The coefficient $b_{gs}$ links two momentum balance equations, namely Equations (28) and (29). It forms the interchange force, $b_{gs}\left(v_{g}-v_{s}\right)$, between the solid and fluid and vice versa. In this particular case, when the solid velocity is equal to zero ($v_{s}=0$), the expression $b_{gs}\left(v_{g}-v_{s}\right)$ is reduced to the Darcy–Forchheimer slip resistance force $f_{D-F}$. From the above considerations, $b_{gs}$ must be dependent on the following parameters:

(43) $$b_{gs}=b_{gs}\left(\varepsilon ,a,\tau ,\mu ,\mu^{\prime \prime },\;\ldots ,\left|v_{g}\right|,p_{g}\right)$$

which includes the porosity, surface density, tortuosity, bulk and surface viscosity, slip speed $\left|v_{g}\right|$, and other surface contributions. The bulk viscosity $\left(\mu \right)$ and surface viscosity $\left(\mu^{\prime \prime }\right)$ appear together as the Navier slip length $l_{s}=\mu /\mu^{\prime \prime }$. Alternatively, the surface viscosity together with the surface density, a, form a physically measurable coefficient, $K_{1}=f\left(\mu^{\prime \prime },a\right)$, which is called the permeability coefficient in the literature (sometimes denoted by the letter κ). Physically, the permeability coefficient describes the interactions between the surface viscosity and the whole control volume. Historically, the first scientist who measured this coefficient was Henry Darcy (1856) [71].

Another factor is the tortuosity, τ, which is strongly related to the slip velocity, $\left|v_{g}\right|$, via the second permeability coefficient, $K_{2}=f(\tau ,$ $\left|v_{g}\right|,\;a)$, also known as the inertial permeability or the Forchheimer coefficient. The importance of this parameter depends on the type of flow. In the case of slow flow in the middle of a a sandstone reservoir, the Forchheimer equation is usually not needed, but in the case of gas inflow into a gas production well, the velocity may be high enough to justify the use of the Forchheimer correction. Some carbonate porous bodies have many internal fractures, so the Darcy equation for multiphase flow is generalized in order to govern both flows, i.e., in the fractures and in the matrix (i.e., traditional porous rock). The irregular surface of the fracture walls and the high flow rate in the fractures may also justify the use of the Forchheimer equation.

Another key parameter that is helpful in describing volumetric momentum transport is the Duhem slip force, $f_{r}^{Du}=p_{g}\mu^{0}v_{g}/\left|v_{g}\right|$, which is a part of the gas resistivity ($\mu^{0}$ is the Duhem surface static-like viscosity [54]).

The Klinkenberg correction to the Darcy permeability, $K_{1}$, is defined as:

(44) $$K_{1}^{eff}=K_{1}\left(1+\frac{b}{p_{g}}\right)$$

where the collective influence of the Duhem resistivity, $\mu^{0}$, is now represented by the Klinkenberg parameter. Finally, the volumetric momentum transport is represented via the following formula:

(45) $$b_{gs}=\frac{\mu }{K_{1}\left(1+b/p_{b}\right)}\;\varepsilon +\frac{\mu }{K_{2}}\;\varepsilon^{2}\left|v_{g}\right|$$

$K_{1}$ is measured in m^2^. An additional important factor is the hydraulic conductivity coefficient, $K=K_{1}\rho g/\mu$, which is measured in m/s (see Sobieski and Trykozko [17]).

### 6.3. Volumetric Heat Transport

The key parameter governing the volumetric heat exchange is the coefficient $h_{cat}$, which was already used in Equations (35) and (37). This coefficient can be defined on the basis of the surface heat transport equation, Equation (24):

(46) $$h_{cat}=a_{v}h\;\mathrm{and}\;a_{v}=169.4\;PPC$$

where h is the surface heat transfer coefficient (between the gas mixture and the solid), $a_{v}$ is the cross-sectional area related to the unit volume of the porous material, and PPC (pores per centimeter) is the number of pores per centimeter in the length of the porous medium. Please note the difference between the convective heat transfer coefficient h and the volumetric heat transfer coefficient $h_{cat}\;$(see Nomenclature).

## 7. Constitutive Relationship for Bulk Properties

For fluids, the equation governing viscous Newtonian gas mixtures is given by (Badur and Ochrymiuk [8]):

(47) $$\tau_{g}=-\frac{2}{3}\mu I_{d}I+2\mu d$$

where μ is the gas mixture viscosity. The Fourier-like diffusional flux of thermal energy is given by:

(48) $$q_{g}=\lambda_{g}g_{g}$$

The heat diffusion in the gas mixture is governed by the total diffusion coefficient, $\lambda_{g}$ (Badur and Ochrymiuk [8]), and the gradient of the gas temperature is given by $g_{g}=\mathrm{grad}\;T_{g}$.

For solids, the heat flow is enhanced, since a collective mode of heat transport appears in the Fourier relation:

(49) $$q_{s}=\lambda_{s,eff}g_{s}$$

The heat diffusion in the solid is enhanced via additional contributions due to the gas flow velocity $v_{g}$ and is given as (Sobhan and Peterson [72]):

(50) $$\lambda_{s,eff}=\lambda_{s}+\left|v_{g}\right|\;d\;c_{p,g}K_{1}^{-1}$$

However, the radiation energy source is given by the classical Stefan–Boltzmann formula (Weisz [14]):

(51) $${\dot{Q}}_{rad}=\left(1-\varepsilon \right)\;\alpha \;k_{B}\left(T_{s}^{4}-T_{0}^{4}\right)\exp\left(-\frac{\psi x}{l}\right)$$

where $k_{B}$ is the Stefan–Boltzmann constant, α is the emissivity of the solid, ψ=−lnε is the extinction function, and x/l is the dimensionless position of the radiating place. The temperature of the environment was determined to be $T_{0}=288^{o}\;K$.

## 8. Terzaghi Concept of Effective Stresses in Porous Solids

The concept of mechanical coupling between fluids and porous solids has been recognized for over a century, as the first significant concept of FSIs combining models appeared in the 1920s. In Europe, Karl Terzaghi developed two crucial ideas, namely the notions of effective stress and the diffusion of fluid pressure due to flow. The classical Terzaghi concept of effective stress can be stated as (Kubik and Cieszko [71]):

(52) $$\sigma =\sigma^{\prime }+\alpha p_{g}I=\left(\sigma_{jk}^{\prime }+\alpha p_{g}\delta_{jk}\right)e_{j}⨂e_{k}$$

which is a constitutive equation of porous material. It proposes to decompose the elastic formulae of the stress tensor to account for the joint properties of the solid and surrounding fluid. Then, if:

(53) $$\sigma_{jk}^{\prime }=2G\varepsilon_{jk}+K_{s}\varepsilon_{mm}\delta_{jk}$$

is the classical Hooke law for a linearly elastic material, a change in the volumetric effective stress is given by three parameters: $G,\;K_{s},\;and\;p_{g}$. In Equation (52), the correcting coefficient, α, takes into account the influence of “pore rigidity” in the Biot form (Kubik and Cieszko [71]) $\alpha =1-K/K_{s}$ (where K is the bulk modulus of the porous medium and $K_{s}$ is the bulk modulus of the solid grains). The change in the fluid pressure is denoted by p. The equation accurately describes the behavior of rocks under laboratory conditions. As the porosity approaches zero, K gets closer to $K_{s}$, so the influence of the pore pressure on the effective stress vanishes as expected [17].

Under the assumption that the deformation of solid continua is “additive”, the linearized strain tensor $\varepsilon =\frac{1}{2}\;\left(\mathrm{grad}\;u+{\mathrm{grad}}^{T}u\right)=\varepsilon_{kl}e_{k}⨂e_{l}$ can be composed as a sum of particular independent contributions, such as elastic, porous, thermal, and gas sorption:

(54) $$\varepsilon =\varepsilon^{el}+\varepsilon^{po}+\varepsilon^{th}+\varepsilon^{so}=\;=\varepsilon^{el}+\frac{\alpha }{K}\;\left(p-p_{0}\right)I+\beta \left(T-T_{0}\right)I+\overset{m}{\underset{b=1}{\sum }}\beta_{\left(b\right)}\left(Y_{\left(b\right)}-Y_{0\left(b\right)}\right)I$$

This means that elastic, porous, thermal, or gas sorption stresses cannot be indicated separately. Therefore, it was assumed that there are the following constitutive relations for isotropic materials between the total stress and total deformation tensors:

(55) $$\sigma =2G\varepsilon +2G\frac{\nu }{1-2\nu }tr\left(\varepsilon \right)I+\alpha \left(p-p_{0}\right)I+\;+2G\frac{1-\nu }{1-2\nu }\left[\beta_{T}\left(T-T_{0}\right)+\overset{m}{\underset{\left(b\right)=1}{\sum }}\beta_{\left(b\right)}(Y_{\left(b\right)}-Y_{0\left(b\right)}\right]I$$

This is in a form that is convenient for direct use in Equation (6) as div σ. In the above, G and v are the Kirchhoff and Poisson coefficients for solid (non-porous) materials, α is the Biot coefficient $\alpha =1-K/K_{s}$ (Kubik and Cieszko [71]) (where K is the bulk modulus of the porous medium and $K_{s}$ is the bulk modulus of the solid grains), $\beta_{T}$ is the thermal expansion coefficient for porous material, and $\beta_{\left(b\right)}$ is the coefficient of gas sorption. The reference pressure, temperature, and mass fraction, i.e., $p_{0},\;T_{0},\;and\;Y_{0\left(b\right)}$, should be known beforehand.

## 9. Terzaghi Concept of Effective Stresses in Fluids

The physical closure for $\sigma_{g}$ needs to be discussed. This additional gas transport flux is related to the simplest phenomena of solid-body-like deformations of pores:

(56) $$\sigma_{g}=a^{-2}\rho_{g}{\left(s\right)}^{2}\mathrm{grad}\;I_{\varepsilon }⨂\mathrm{grad}\;I_{\varepsilon }$$

The conclusion from this is that the effective stresses in a fluid are not in the form of a spherical tensor (which is unusual in fluid constitutions), and it consists of shearing components such as $\sigma_{xy}$. In above equation, a is the surface density, $\rho_{g}$ is the gas moisture density, s is the Stefan slip velocity, and $I_{\varepsilon }=\mathrm{div}\;u$ is the first invariant of the solid deformation tensor.

## 10. Terzaghi Effective Stresses and a Concept of Material Effort

Elasto-plastic and visco-elastic strains occur in the composite material of a burner. Different modes of plastic yielding are observed in composites during constant strain tests. Whether a composite experiences plastic yielding or suffers brittle failure depends on the stress state, temperature, and chemical environment. Plastic deformation can occur as combination of a shear and a volumetric strain, as seen in the study by Kubik and Cieszko [71] and Shi et al. [40]. Visco-elastic behavior (also called creep or time-dependent deformation in composite mechanics) can be very important in in-service operation during start-up and shutdown. Then, the constitutive relationship in Equation (52) must be extended to include the additional contribution of plastic and viscous strains.

A key challenge with elasto-plasticity (and elasto-viscosity) is the specification of a material effort by defining a yield surface that describes the boundary between the elastic and plastic state of a material. This problem has been well approached in terms of the principal stresses, $\sigma_{1},\sigma_{2},\;and\;\sigma_{3}$, of the stress tensor, $\sigma =\sigma_{1}t_{1}⨂t_{1}+\sigma_{2}t_{2}⨂t_{2}+\sigma_{3}t_{3}⨂t_{3}$, which are the largest, intermediate, and lowest principal stresses and are related to the principal axes $t_{1},t_{2},\;and\;t_{3}$. Experimental results take into account the total strength, so in order to obtain the effective Terzaghi stresses, $\sigma^{\prime }$, carried by the skeleton, the pore pressure needs to be subtracted from the total stress tensor, σ. The basic approach is to treat the yield surface as a Mohr–Coulomb failure envelope, which predicts yielding when the effective principal stresses deviate sufficiently from a hydrostatic state. A more comprehensive approach is provided by the critical state theory, which postulates a yield surface dependency on the effective stresses, strain state, and load history.

Wound ceramic matrix composites (CMC) are built from ceramic fibers embedded in a ceramic porous matrix [40]. They have many advantages over classical materials due to their high oxidation resistance, graceful failure behavior, thermal shock resistance, and sufficient strength at elevated temperature. These properties exceed all other materials used in porous burner technology. The concept of material effort within such strongly anisotropic porous materials is very complex and is based on a general hypothesis proposed by von Mises (1928) [40]:

(57) $$f=\sigma_{ij}^{\prime }F_{ij}+\sigma_{ij}^{\prime }\sigma_{kl}^{\prime }F_{ijkl}-1=0\;\mathrm{for}\;\left(i,j,k,l=x,y,z\right)$$

This concept of material effort is based on effective Terzaghi stresses; therefore, the strength limits, $F_{ij}$ (second-order tensor) and $F_{ijkl}$ (fourth-order tensor), should be experimentally realized within the proper pressure conditions [40]. Analyzing the ceramic composite WHIPOX has resulted in the determination of five independent limits, $F_{1},F_{2},F_{11},F_{22},\;and\;F_{66}$, and has reduced (57) to the well-known Tsai-Wu hypothesis. Unfortunately, their experiments concerned only atmospheric pressure, where $p_{g}=p_{atm}$.

## 11. The Boundary Conditions

In the most general case, a porous combustion chamber is in the form of a long cylinder (Groppi et al. [11]) with circular inlet (z=0) and outlet (z=l) sections. Based on a given inlet temperature $T_{g,in}$, inlet mixture pressure $p_{g,in}$, outlet mixture pressure $p_{g,out}$, and mixture content, the mass flow rate at the inlet can be calculated as $\dot{m}=\iint \rho v_{g}\cdot n\;dA$. Additionally, the radiative inlet conditions for the solid ($q_{s,z=0}\cdot n=q_{s,rad}$) should be correctly predicted. At the initialization step, the temperature of the catalytic layer can be set to be equal to the gas temperature, i.e., $T_{cat,in}=T_{g,in}$, and the catalytic density can be set based on the reference, out–off reaction density $\rho_{cat,in}=\rho_{cat,ref}$.

## 12. The Rate of Disaggregated Energy Production

The Bejan concept of disaggregated energy production [34] (similar to the Feidt concept [35,36,44]) for the case of catalytic combustion, includes the explanation of key assumptions, such the whole production of local entropy. It depends on the generation of entropy due to heat, friction, diffusion, and chemical contributions:

(58) $${\dot{E}}_{gen}={\dot{E}}_{gen,heat}+{\dot{E}}_{gen,\;fric}+{\dot{E}}_{gen,\;diff}+{\dot{E}}_{gen,\;chem}$$

In the above, ${\dot{E}}_{gen}$ (J s^−1^) is the whole-domain (integral) energy rate disaggregation, which is produced over the whole computational domain. In a cylindrical coordinate system, ${\dot{E}}_{gen}$ for a cylindrical domain is given as:

(59) $${\dot{E}}_{gen}=\iint 2\pi r\;{\dot{\epsilon }}_{gen}\;drdx$$

where r and x are the radial and axial coordinates, respectively. The following components of disaggregated energy production were adopted from the literature:

- Generation due to the heat diffusional flow:

(60) $${\dot{\epsilon }}_{gen,heat}=T_{g}^{-2}\;\lambda_{g}[{\left(\partial_{x}T_{g}\right)}^{2}+\left(\partial_{r}T_{g}{)}^{2}\right]+H{\left(T_{s}-T_{g}\right)}^{2}$$

- Generation due to viscous bulk and surface friction:

(61) $${\dot{\epsilon }}_{gen,fric}=T_{g}^{-1}\mu \;\Phi +f_{D-F}\cdot s\;\mathrm{and}\;\Phi =2d\cdot d$$

- Production due to species mixing:

(62) $${\dot{\epsilon }}_{gen,diff}=\sum_{i=1}^{N_{S}}\rho D_{mi}\;J_{i}\cdot J_{i}+\sum_{\left(b\right)=1}^{m}\rho_{cat}D_{m\left(b\right)}\;j_{\left(b\right)}\cdot j_{\left(b\right)}$$

- Production due to catalytic chemical reactions:

(63) $$\;{\dot{\epsilon }}_{gen,chem}=\sum_{i=1}^{N_{S}}Y_{i}h_{i}{\dot{\omega }}_{i}+\overset{m}{\underset{\left(b\right)=1}{\sum }}{\dot{\omega }}_{\left(b\right)}W_{\left(b\right)}h_{\left(b\right)}$$

The above relations can be computed after the simulation in a post-processing stage.

## 13. An Example of Heat Transfer Analysis Using Thermal FSI

**Figure 5:**
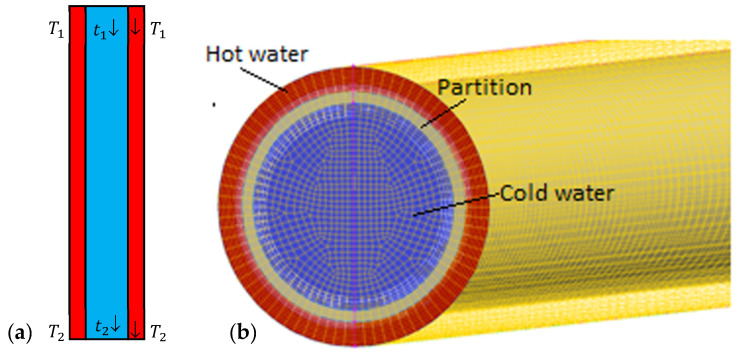
Stanton benchmark experiment; (**a**) cross-section, (**b**) numerical model.

The elements of the above multi-field model have been verified within the scope of different publications (Badur et al. [7], Badur and Ochrymiuk [8], Lewandowski et al. [73], Ochrymiuk [27,27], Ziółkowski et al. [43], Ziółkowski and Badur [33]). As an example, its application can be demonstrated with a simple thermal FSI benchmark example of the Stanton experiment (Stanton [74]), which was prepared in order to prove the Reynolds mechanism of thermal energy transport (Reynolds [56], Stanton and Pannell [75]). This is a simple case, where heat flux flows across the cylindrical metal wall from the hot water stream ($T_{1}=$ 39.6 °C) towards a co-flowing cold water stream ($t_{1}=$18 °C), as shown in Figure 5.

The total length of pipes was 67 cm, and the internal diameters were 13.9 mm (cold) and 15.7 mm (hot) (Stanton [74]). The mass flux of hot water was constant during the experiment and was equal to 148 g/s, whereas the mass flux of cold water varied from 148 g/s to 27 g/s. Note that the cold water inlet temperature was kept constant.

The aim of the Stanton experiment was to demonstrate the role of turbulent heat transfer. For the same mass flux of 148 g/s of hot and cold water, the result was symmetrical due to the conservation of energy, i.e., an increase in the temperature of the cold water by 4.23 °C ($t_{1}=18\;$°C → $t_{2}=22.23\;$°C) induced at temperature drop in the hot water by the same amount ($T_{1}=39.6\;$°C → $T_{2}=35.37\;$°C). This result was consistent between the experiment and simulation (see Table 1). The cold water mass flux reduction from 148 g/s to 27 g/s resulted in a systematic increase in the outlet temperature, $t_{2}$, from 22.23 °C to 23.25 °C and, simultaneously, an increase in $T_{2}$ from 35.2 °C to 37.4 °C.

It is worth mentioning that quite similar results have been numerically obtained by implementing the Reynolds–Stanton analogy (Reynolds [76]). It was calculated that during the change in the mass flow rate of the cold water (average velocity change from 24.4 to 4.5 cm/s), the total exchange of energy due to heating increased from $\dot{H}=$ 336 W to $\dot{H}=$ 390 W and harmonized with the formulae proposed by Reynolds and empirically proved by Stanton (in an original notation):

(64) $$\dot{H}=\left(A+B\rho c\right)\left(T_{1}-t_{1}\right)$$

with A=343 and B=334, as calculated by Stanton and repeated in thermal FSI from an overall integration of the normal component of the heat vector:

(65) $$\dot{H}=\iint q\cdot n\;dA$$

It has been shown by numerical simulation that the heat flux (Equation (64)) depends directly on the length of the heat exchanging pipe; therefore, the Reynolds–Stanton formulae should be replaced by:

(66) $$\dot{H}=l\left(518+499\rho c\right)\left(T_{1}-t_{1}\right)$$

where l is the length of the heat exchange pipe. It is also important that the heat flux, $q=q^{l}+q^{t}$**,** was very sensitive to the turbulent heat flux modelling method, which was in agreement with Karcz and Badur [30] and Rup and Wais [77].

## 14. Conclusions

Everyone agrees that mathematical modelling represents a powerful method that is able to support the design process at every stage of the life cycle of catalytic porous combustors used in gas turbines. Therefore, a mathematical model that is robust and not time-consuming needs to be developed. In the paper, we proposed a two-stage model, the first dealing with a relatively exact description of the catalytic layer. Thus, the equations of mass, momentum, and energy conservation within the boundary of this catalytic layer were proposed and discussed. The basic novelty of this model lies in the detailed modeling of surface geometry and surface kinematics.

In the second stage of modeling, we proposed an averaged “two/three fields equation” for porous catalytic combustors. The concept is based on the volumetric (not surface, so far) coefficients of mass, momentum, and energy exchange.

However, the number of complex physical and chemical phenomena affecting the performance of a catalytic combustor is so large that the identification and application of certain approximations is inevitable. Therefore, in the numerical example, one possibility for identifying the parameters of the model was shown. The thermodynamical framework of the model was also opened to further research, i.e., the Bejan approach can be adopted.

The literature contains numerous models, the most popular being direct numerical simulation (DNS), large eddy simulation (LES), the lattice Boltzmann model (LBM), density functional theory (DFT), molecular dynamics (MD), and the Monte Carlo (MC) method. In opposition to these, in the present paper, we developed a model based on the concept of fluid–solid interactions. The proposed model has some advantages. It is based on the number of zones taken into consideration (fluid, solid, and catalytic layer). Depending on this, the proposed model can be described as a two-mass (Equations (25) and (26)), two-momentum (Equations (28) and (29)), and three-temperature model (Equations (35), (37), and (38)). Finally, the key aspects of the different phenomena occurring at the catalyst section were assessed by comparing simulation results.

## Figures and Tables

**Table 1:** Comparison of the experimental and numerical outlet temperature, $t_{2}$.

Cooling Water Mass Flow [g/s]	Velocity of Cooling Water [cm/s]	Stanton $t_{2}$ [°C]	CFD $t_{2}$ [°C]
148	98	22.23	21.72
104	69	22.45	22.17
88	58.1	22.60	22.47
66	43.6	22.76	23.25
43	28.6	23.05	24.71
27	18	23.25	23.59

## Nomenclature

Fluid in the bulk	
$\rho_{g}$	mass density of gas [kg m^−3^]
$Y_{i}$	mass fraction of species i in the bulk mixture
[$X_{i}$]	molar concentration of ith species
$N_{R}$	chemical reactions
$W_{i}$	molar density of each species
${\dot{\omega }}_{i}$	rate of production of ith species
$v_{ki}^{\prime }$, $v_{ki}^{\prime \prime }$	stoichiometric coefficients of ith species and kth reacton
$k_{k}^{f},\;k_{k}^{r}$	forward and reverse rate constant for the kth reaction
$E_{k}$	activation energy of kth reaction
$A_{k},\;\beta_{k}$	stoichiometric coefficients in the Arrhenius expression
$J_{i}$	Dixon–Levis flux of ith species mass
R	gas constant of mixture
$h_{i}$	total enthalpy of ith species
$t_{g}$	Cauchy momentum flux in the gases mixture
Catalytic surface layer	
β	site density (2.7063 × 10^−5^ [mol m^−2^] for Pt)
$\varphi_{\left(b\right)}$	fraction of surface sites covered by species $Y_{\left(b\right)}$
$A_{cat}$	heterogeneous surface reaction area
$A_{geo}$	geometrical surface area
$\varepsilon_{s}$	surface porosity (Equation (19))
$\rho_{cat}$	the surface density [kg m^−2^] of catalytic layer
$\left(X_{\left(b\right)}\right)$	the mole surface concentration [mol m^−2^]
$Y_{\left(b\right)}$	mass fraction of species $\left(b\right)$ within the catalytic layer [kg kg^−1^]
m	number of gas-phase species on the catalytic layer
$N_{s}$	number of surface reactions
$W_{\left(b\right)}$	the molecular mass of species $\left(b\right)$. [kg mol^−1^]
${\dot{\omega }}_{\left(b\right)}$	net production rate of species $\left(b\right)$ due to surface reactions [mol m^−2^ s^−1^]
$\nu_{\left(b\right)p}$, $\nu_{\left(b\right)p}^{\prime }$	stoichiometric coefficients, bth product species, pth th reaction
$k_{p}$	rate coefficient of pth product species
$\xi_{\left(b\right)}$; $e_{\left(b\right)}$	two additional coverage factors
$A_{p}$	pre-exponential factor
$\beta_{p}$	temperature exponent
$E_{p}$	activation energy
$j_{\left(b\right)}$	surface diffusive flux [kg s^−1^ m^−2^]
s	Stefan slip velocity [m s^−1^]
n	normal to the catalytic surface, $a_{\alpha }$, α=1, 2 surface coordinate base
$T_{cat}$	temperature of catalytic layer [K]
Σ	middle surface of the catalytic layer
$t_{cat}$	surface excess of flux of momentum (Equation (4))
$I_{2}$	surface metric tensor
$II_{2}$	surface curvature tensor
$d_{2}$	surface rate of deformation
$\mu_{2}$	Navier surface viscosity
$a_{2}$	d’Alembert-Euler surface acceleration (Equation (5))
$f_{r}=f_{r}^{Du}+f_{r}^{Na}+f_{r}^{Bu}$	surface resivitivity forces (Equation (14))
$f_{m}=f_{m}^{Ga}+f_{m}^{Re}+f_{m}^{Ma}+\ldots$	surface mobility force (Equation (14))
Porous solid continuum	
$\rho_{s}$	mass density of solid (kg/m^3^)
$T_{s}$	solid temperature [K]
$\sigma_{s}$	Cauchy stress tensor,
$\sigma^{\prime }$	Terzaghi stress tensor
ε	strain tensor
Others	
${\dot{E}}_{gen}$	disaggregated energy production (Equation (58))
